# HCV 6a Prevalence in Guangdong Province Had the Origin from Vietnam and Recent Dissemination to Other Regions of China: Phylogeographic Analyses

**DOI:** 10.1371/journal.pone.0028006

**Published:** 2012-01-09

**Authors:** Yongshui Fu, Weibing Qin, Hong Cao, Ru Xu, Yi Tan, Teng Lu, Hongren Wang, Wangxia Tong, Xia Rong, Gang Li, Manqiong Yuan, Chunhua Li, Kenji Abe, Ling Lu, Guihua Chen

**Affiliations:** 1 Department of Biochemistry, Medical College of Sun Yat-sen University, Guangzhou, Guangdong, China; 2 Guangzhou Blood Center, Guangzhou, Guangdong, China; 3 Family Planning Research Institute of Guangdong Province, Guangzhou, Guangdong, China; 4 The Vaccine Institute, Third Affiliated Hospital of Sun Yat-sen University, Guangzhou, Guangdong, China; 5 Department of Infectious Diseases, Third Affiliated Hospital of Sun Yat-sen University, Guangzhou, Guangdong, China; 6 Fogarty International Center, National Institutes of Health, Bethesda, Maryland, United States of America; 7 University of Southern California, Los Angeles, California, United States of America; 8 Department of Pathology and Laboratory Medicine, Viral Oncology Center, University of Kansas Medical Center, Kansas City, Kansas, United States of America; 9 Department of Pathology, National Institute of Infectious Diseases, Shinjuku-ku, Tokyo, Japan; 10 Laboratory for Hepatology, Third Affiliated Hospital of Sun Yat-sen University, Guangzhou, Guangdong, China; Tsinghua University, China

## Abstract

**Background:**

Recently in China, HCV 6a infection has shown a fast increase among patients and blood donors, possibly due to IDU linked transmission.

**Methodology/Findings:**

We recruited 210 drug users in Shanwei city, Guangdong province. Among them, HCV RNA was detected in 150 (71.4%), both E1 and NS5B genes were sequenced in 136, and 6a genotyped in 70. Of the 6a sequences, most were grouped into three clusters while 23% represent emerging strains. For coalescent analysis, additional 6a sequences were determined among 21 blood donors from Vietnam, 22 donors from 12 provinces of China, and 36 IDUs from Liuzhou City in Guangxi Province. Phylogeographic analyses indicated that Vietnam could be the origin of 6a in China. The Guangxi Province, which borders Vietnam, could be the first region to accept 6a for circulation. Migration from Yunnan, which also borders Vietnam, might be equally important, but it was only detected among IDUs in limited regions. From Guangxi, 6a could have further spread to Guangdong, Yunnan, Hainan, and Hubei provinces. However, evidence showed that only in Guangdong has 6a become a local epidemic, making Guangdong the second source region to disseminate 6a to the other 12 provinces. With a rate of 2.737×10^−3^ (95% CI: 1.792×10^−3^ to 3.745×10^−3^), a Bayesian Skyline Plot was portrayed. It revealed an exponential 6a growth during 1994–1998, while before and after 1994–1998 slow 6a growths were maintained. Concurrently, 1994–1998 corresponded to a period when contaminated blood transfusion was common, which caused many people being infected with HIV and HCV, until the Chinese government outlawed the use of paid blood donations in 1998.

**Conclusions/Significance:**

With an origin from Vietnam, 6a has become a local epidemic in Guangdong Province, where an increasing prevalence has subsequently led to 6a spread to many other regions of China.

## Introduction

Because of frequent blood exposures and unsafe injection behaviors, hepatitis C virus (HCV) infection has become highly prevalent among injection drug users (IDU) in China [1]–[4]. Based on a recent meta-analysis, the pooled HCV prevalence among IDUs was about 67.6%, and there were large geographic variations in genotype distribution. Generally, 6a was predominant, accounting for 36.7%. This was followed by 3b, 1a, 3a, and 1b, accounting for 16.4%, 14.6%, 12.8%, and 12.4%, respectively [5]. Among IDUs in the Guangxi and Hubei Provinces, and in Hong Kong and Taiwan as well, 6a was the main HCV strain [5]. However, this was not consistent with the predominant strains among clinical patients. The high proportion of 6a among IDUs in Guangxi has been linked to the fact that HCV was transmitted through unsafe injection by users along the drug trafficking route from Southeast Asia [6].

The Guangdong Province is located in the southern part of China, neighboring Guangxi. Historically, it has been an internal and international crossroads for cultural and commercial exchanges. Since 1978, Guangdong has been assigned as the first region of China to adopt a free market economy and open door policy. Recently, as economic development in China accelerates, this province has become a “World Production Center”. A powerful economy has stimulated a large population of drug users and a rapid increase in drug trafficking and trading. In addition, there has been an explosion of the illegal sex industry in the province and this has been associated with millions of migrants who are on business or pleasure or temporarily hired as laborers in the myriad of factories that lack good hygiene conditions [7]. Collectively, these factors have created an environment to inbreed HCV prevalence.

Recently, we have studied HCV infection among patients and blood donors in the Guangdong Province and found that 6a has accounted for an increasing proportion [8]–[9]. We tend to believe that this 6a prevalence could have resulted from IDU linked transmission. In our phylogenetic analysis, 6a appeared to show its origin from Vietnam, because Vietnamese isolates were the most diverse and genetically distant. Considering the historical event in which the Guangdong, Guangxi, and Hainan provinces had accepted approximately 300,000 refugees from Vietnam during the late 1970 s [9], we hypothesized that 6a may have since been introduced into Guangdong and become a local epidemic to seed the IDU network. In recent decades, 6a has been spread to other regions by IDUs or by infected people among the migrants. In this study, coalescent and phylogeographic analyses were performed to test this hypothesis.

## Results

### Prevalence of anti-HCV and HCV RNA

For routinely screening blood donations, an automated, integrated NAT (Nucleic Acid Testing) platform has been implemented at the Guangzhou Blood Center. Therefore, samples from this study were first applied to this platform. After testing for HCV RNA, the samples were subjected to PCR. This resulted with only 198 samples left for anti-HCV assay. Consequently, 191/198 (94.5%) users were anti-HCV positive and 150/210 (71.4%) were HCV RNA positive (Table 1).

**Table 1:** Positive rates of anti-HCV and HCV RNA among drug users.

		Anti-HCV			HCV RNA		
		Total	Positive	%	Total	Positive	%
Age	<20	6	6	100	6	2	33.3
	21–30	62	60	98.4	68	46	67.7
	31–40	109	104	95.4	114	82	71.9
	41–50	18	18	100	18	16	88.9
	>50	3	3	100	4	4	100
Gender	Male	188	181	96.2	200	142	71.0
	Female	10	10	100	10	8	80.0
Marriage	Married	82	78	95.1	86	60	69.8
	Not married	116	113	97.4	124	90	72.6
Origin	Guangdong	186	180	96.8	198	142	71.7
	Not Guangdong	12	11	91.7	12	8	66.7
Education	≥High school	38	37	97.4	40	29	72.5
	Middle school	77	74	96.1	83	60	72.3
	≤Elementary	83	80	96.4	87	61	70.1
Job	Jobless	103	99	96.1	109	77	70.6
	Had a job	95	92	96.8	101	73	72.3
Surgery history	Yes	38	37	97.4	42	33	78.6
	No	160	154	96.3	168	117	69.6
Transfusion history	Yes	21	20	95.2	22	16	72.7
	No	177	171	96.6	188	134	71.3
Injection drug use	Yes	176	172	97.7	185	144	77.8
	No	22	19	86.4	25	6	24.0
Years of drug use	<2	48	47	97.9	49	31	63.3
	2–5	30	29	96.7	35	21	60.0
	6–10	68	66	97.1	72	55	76.4
	>10	52	49	94.2	54	43	79.6
Years of injection	<1	40	40	100	41	33	80.5
	1–5	45	42	94.4	50	40	80.0
	6–10	47	46	97.9	49	40	81.6
	>10	44	44	100	45	37	82.2

### HCV sequence amplification and phylogenetic analysis

Of the 150 users positive for HCV RNA, both E1 and NS5B genes were amplified in 136 (90.7%) but failed in 14 (9.3%). Thus, HCV was genotyped: 6a in 70 (51.5%), 3a in 42 (30.9%), 1b in 13 (9.6%), 3b in 3 (1.6%), and multiple HCV infections in 8 users (5.9%) (Table 2).

**Table 2:** HCV genotype distribution.

	Years of age				Gender		By injection		Years of drug use		Needle sharing	
	≤30	31–40	≥41	Mean ± SD	Male	Female	Yes	No	≤2	>2	Yes	No
6a (n = 70)	30	32	8	32.8±7.6	67	3	61	9	25	45	34	36
3a (n = 42)	12	25	5	34.1±6.2	40	2	36	6	9	33	19	23
1b (n = 13)	2	9	2	35.3±8.3	13	0	13	0	4	9	7	6
3b (n = 3)	1	1	1	35.3±8.5	3	0	3	0	1	2	1	2
Multiple (n = 8)	4	3	1	32.0±6.9	8	0	8	0	3	5	6	2

1b sequences were isolated from 13 users. Among them one was classified into a previously designated “A” cluster, three into a “C” cluster [9], and one was grouped with reference Chinese isolates. In contrast, eight formed their own cluster designated “SW”. The SW had a bootstrap score of 95% in the E1 and 89% in the NS5B tree (Figure S1).

3a sequences were isolated from 42 users. They formed a monophyletic cluster in both the E1 and NS5B trees (Figure S2). Genetically, they looked distinct from those isolated from IDUs in the Hubei [2] and Yunnan Provinces [10] but with no significant bootstrap support.

**Figure 1:**
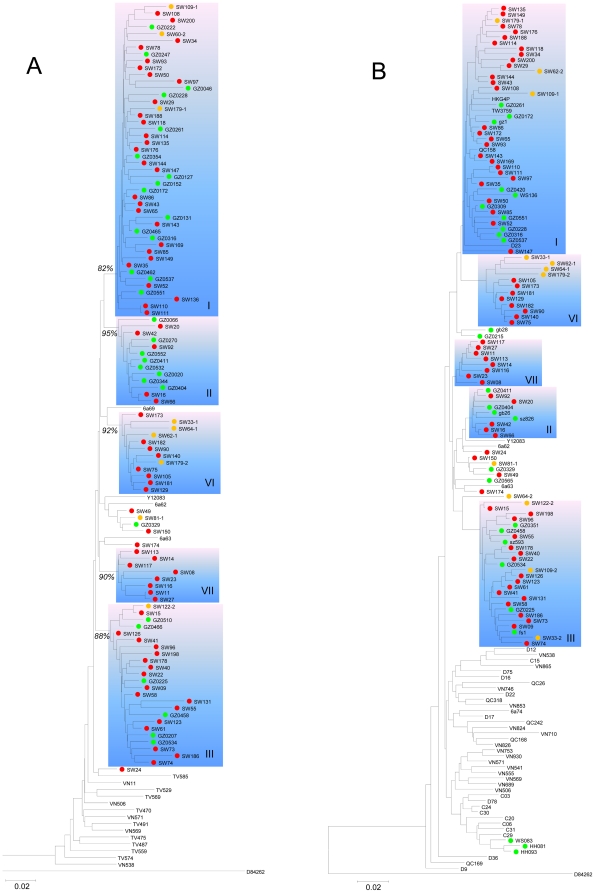
Subtype 6a phylogenies estimated from (A) E1 and (B) NS5B region sequences, corresponding to H77 nucleotide positions of 869–1289 and 8276–8615, respectively. Subtype 6b sequence D84262 was used as an outgroup. Green pies label sequences from our previous studies [8], [9]. Red and yellow pies label sequences from this study, in which yellow pies mark isolates from IDUs with multiple HCV infections. Sequences without pies were retrieved from Genbank. In each tree, five rectangles highlight the further classification of 6a isolates into I, II, III, VI, and VII clusters. Scale bar represents 0.02 nucleotide substitutions per site.

6a sequences were isolated from 70 users. Among them, 28, 5, and 17 were classified into clusters I, II, and III; 8 each were grouped into clusters VI and VII (Figure 1). Clusters I, II, and III have been recently described and included many reference sequences [2], [3], [6], [8], [9]. Clusters VI and VII were newly designated and contained sequences only from this study (accounting for 23%). Both the E1 and NS5B sequences were analyzed and classified isolates consistently. Therefore, identical isolates were placed in similar positions in both trees. The E1 tree further showed bootstrap scores of 82%, 95%, 88%, 92%, and 90% for clusters I, II, III, VI, and VII, respectively. However, no significant scores were shown in the NS5B tree.

### Multiple HCV infections

Directly sequencing amplicons yielded ambiguous results for eight users. Molecular cloning followed by sequencing revealed that they had multiple infections with different HCV strains. This was confirmed by sequencing both E1 and NS5B genes from two users, sequencing both core and E1 from one, and sequencing single NS5B from five. SW33, SW62, SW64, SW109, and SW179 each had co-infection with two 6a strains, of which SW64 and SW109 also had a third strain of 1b. SW122 had co-infection with 6a and 1b, while SW60 and SW81 had co-infection with 6a and 3a (Table 3).

**Table 3:** Multiple HCV infections among 8 drug users.

ID	NS5			E1			Core*		Injectionuser	Sharingequipments	Years ofdrug use
	No. clones	Genotype	clusters/close¶	No. clones	Genotype	clusters/close¶	No. clones	Genotype			
SW33	2	6a	VI	6	6a	VI			Yes	Yes	>10 years
	3	6a	III								
SW60	4	3a	SW100	3	3a	SW100	2	3a	Yes	No	<1year
				2	6a	I	2	6a			
SW62	2	6a	VI	5	6a	VI	5	6a	Yes	Yes	6–7 years
	3	6a	I								
SW64	2	6a	VI	5	6a	VI	5	6a	Yes	Yes	10 years
	3	6a	GZ0565								
	2	1b	B								
SW81	2	6a	GZ0329	5	6a	GZ0329	5	6a	Yes	No	<1year
	3	3a									
SW109	2	6a	I	5	6a	I	5	6a	Yes	Yes	10 years
	3	6a	III								
	2	1b	SW								
SW122	4	1b	SW30	4	1b	SW30			Yes	Yes	<1 year
	2	6a	III	2	6a	III					
SW179	2	6a	I	3		I			Yes	Yes	7–8 years
	3	6a	VI	2		VI					

*Genotyping 6a with core region can not differentiate clusters, because of the high similarity of the nucleotides.

¶ Not classifiable into clusters. this isolate was close to a reference.

### Coalescent analyses of 6a sequences

#### (1) 285 dated sequences

Many 6a sequences have been reported among IDUs in China [2], [3], [6], [10]. We have also characterized a number of 6a sequences from patients and blood donors in the Guangdong Province [8], [9]. Jointly, they represented 139 retrieved sequences. Here, 6a sequences were further determined among 21 blood donors from Vietnam, 22 blood donors from 12 provinces of China, and 36 IDUs [3] from Liuzhou City in Guangxi Province. They were co-analyzed with those from 70 IDUs in Shanwei city. In total, 285 isolates were represented, each with 519 nt of E1 gene.

#### (2) Model test

Prior to coalescent analysis [11], model test was performed [12]. Based on BIC (Bayesian information criterion) and AIC (Akaike information criterion), the GTR+I+Γ model was found to be the best. However, based on AICc (Akaike information criterion, corrected), the K80+Γ model was the best. Compared to AIC and BIC, AICc favors simpler models by scoring higher penalties for extra parameters. Since in the BEAST package [13] the model complexity is endeavored and K80+Γ is not implemented, we selected the GTR+I+Γ model.

#### (3) Initial coalescent analyses

Initial coalescent analyses were performed directly with the 285 sequences. After running MCMC (Markov Chains Monte Carlo) for 200 million states under three models, BSP + uncorrelated exponential, BSP + uncorrelated lognormal, and BSP + strict clock, respectively, the mean substitution rates, 1.753×10^−3^ (95% CI: 2.3321×10^−4^ to 3.5632×10^−3^), 2.4472×10^−3^ (95% CI: 1.8745×10^−3^ to 3.1487×10^−3^), and 1.69×10^−3^ (95% CI: 1.1033×10^−3^ to 2.231×10^−3^) per site per year, were estimated. Although the exponential model was shown to be better than lognormal (measured by Bayesian Factor, BF>15) and strict models (BF>72.6), three resulting statistics (posterior, mean.Rate, uced.mean) lacked effective sample sizes (ESS≤20). In addition, it showed two separate peaks of trace distribution for the rate and a high COV (coefficient of variation) (mean = 0.879, 95%CI: 0.8191 to 0.9417), both of which might suggest different substitution rates between the Chinese and Vietnamese lineages, consistent with a previous report [14].

#### (4) Estimating rates as priors

To obtain more valid rates to be used as priors in subsequent analysis for better representing the Chinese isolates, 139 sequences were selected with exclusion of all the Vietnamese strains. The 139 sequences were from our studies to ensure correct sampling dates and provide good temporal and phylogenetic structures. Under three models: BSP + uncorrected exponential, BSP + uncorrected lognormal, and BSP + strict clock, the substitution rates, 2.737×10^−3^ (95% CI: 1.792×10^−3^ to 3.745×10^−3^), 2.0615×10^−3^ (95% CI: 1.4786×10^−3^ to 2.8687×10^−3^), and 7.778×10^−4^ (95% CI: 8.1928×10^−5^ to 1.7478×10^−3^) were estimated. Possibly because a short and recent time interval (2002 to 2009) was covered by the 139 sequences, great differences were observed among the rates. However, sufficient ESS was achieved for all statistics. BF analysis confirmed that the exponential model was better than lognormal (BF = 57.75) and lognormal better than the strict (BF = 8.42).

#### (5) Coalescent analyses using rates as priors

Using the rate 2.737×10^−3^ (95% CI: 1.792×10^−3^ to 3.745×10^−3^, standard error: 1.507×10^−5^) as a prior under the BSP + uncorrected exponential model, a coalescent analysis was performed for the 285 sequences (Table 4). Transforming the log file into trace distributions revealed a substitution rate of 2.7346×10^−3^ (95% CI: 2.7055×10^−3^ to 2.7637×10^−3^), in which the mean is similar to the prior but the 95% CI (confidence interval) is narrower. Parameter α is inversely related to the extent of rate variation among sites [15]. Here, the estimated α was 0.8933 (95%CI: 0.8887 to 0.8978). It is ≤1 and means that most sites have very low rates while mutational “hot spots” existed in the sequences that changed at very high rates. Compared to that previously estimated for core (α = 0.22) and NS5B (α = 0.32) genes [14], a higher α suggests a weaker “hot spot” pattern for the analyzed E1 gene. The pInv statistic shows the proportion of invariable sites and lower value means more variable nucleotides [16]. Here, a moderate pInv 0.4684 (95%CI: 0.4674 to 0.4695) was inferred. COV represents rate heterogeneity among lineages, which abuts zero when a strict molecular clock is followed [17]. For the 285 sequences a high COV, 0.8419 (95% CI: 0.8402 to 0.8437), was estimated. This is higher than 0.37 previously inferred for concatenated core and NS5B genes (95% CI: 0.28 to 0.45) [14] and strongly indicates a relaxed molecular clock. In addition, a very small covariance parameter, 5.652×10^−4^ (95% CI: −0.0011 to 2.2368×10^−3^), was observed. This value measures a degree to which among-lineage rate variation is randomly distributed across the phylogeny rather than being restricted to specific clusters. Using different rates as priors, coalescent analyses were also performed under two other models, BSP + uncorrected lognormal and BSP + strict clock, and the results were detailed in Table 4.

**Table 4:** Statistics generated from three coalescent processes.*

Combination Models	BSP + Exponential¶	BSP + Lognormal‡	BSP + Strict clock†
Posterior	−14904.78(−14987.10∼−14823.27)±1.99	−15029.4017(−15107.40∼−14946.99)±1.66	−15439.89(−15571.56∼−15324.75)±3.47
treeRootHeight	53.42(30.14∼94.00)±1.04	47.95(36.61∼62.19)±4.11×10^−1^	70.40(50.41∼97.38)±5.59×10^−1^
Alpha	0.893(0.889∼0.898)±4.39×10^−5^	0.905(0.899∼0.902)±3.61×10^−5^	0.724(0.616∼0.842)±8.30×10^−4^
PInv	0.468(0.467∼0.469)±7.95×10^−6^	0.468(0.467∼0.470)±1.04×10^−5^	0.398(0.346∼0.450)±3.27×10^−4^
mean.Rate	2.45×10^−3^(2.43×10^−3^∼2.47×10^−3^)±1.31×10^−7^	2.07×10^−3^(1.95×10^−3^∼2.18×10^−3^)±2.54×10^−6^	N/A
uced/ucld/clock.mean	2.735×10^−3^(2.706×10^−3^∼2.764×10^−3^)±1.72×10^−7^	2.062×10^−3^(2.042×10^−3^∼2.082×10^−3^)±1.11×10^−7^	1.690×10^−3^(1.103×10^−3^∼2.231×10^−3^)±1.23×10^−5^
COV	0.842(0.840∼0.844)±9.94×10^−6^	0.547(0.544∼0.549)±1.26×10^−5^	N/A
covariance	5.652×10^−4^(−0.001∼2.237×10^−3^)±8.90×10^−6^	2.944×10^−2^(2.664×10^−2^ ∼3.248×10^−2^)±1.59×10^−5^	N/A
Treelikelihood	−13153.50(−13198.88∼−13110.30)±1.46	−13180.23(−13223.35∼−13133.07)±1.43	−13350.17(−13392.29∼−13309.21)±8.72×10^−1^

*BSPs were estimated through the three processes and shown in Figure 4. BF = 11.371, when the exponential model compared with the Lognormal model; BF = 72.79, when the Lognormal model compared with the strict clock model.

**Figure 2:**
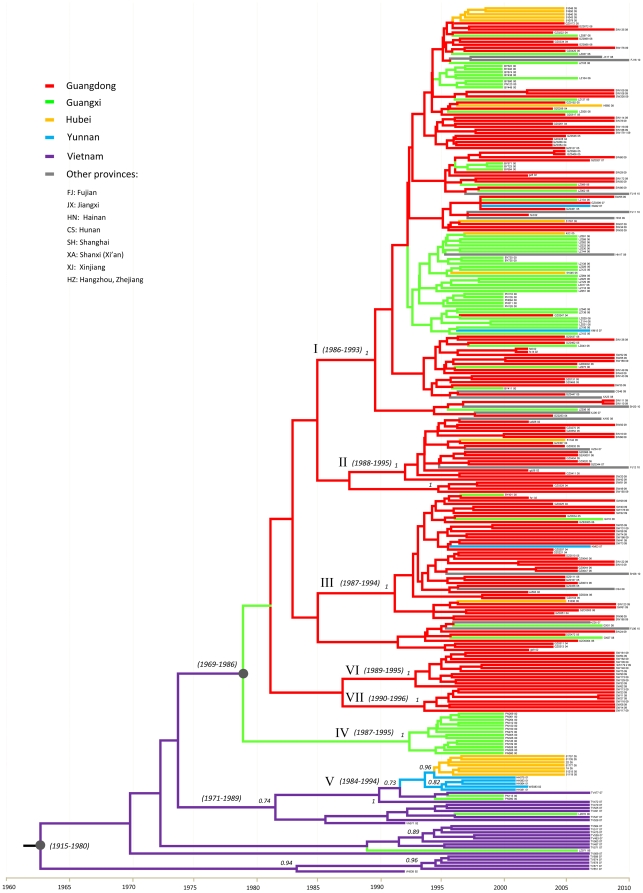
MCC tree estimated under the model of BSP + Uncorrelated Exponential (see **Table 4**). In addition to the isolates presented in Figure 1A, sequences determined among 21 blood donors from Vietnam, 22 blood donors from the 12 provinces of China, and 36 IDUs from Liuzhou City in the Guangxi Province [3] were also included. Branches were colored according to their respective geographic origins. Posterior probabilities greater than 0.9 were shown at the respective nodes, while the estimated time points of the origin for the nodes were parenthesized. Below the tree is a time scale from 1960–2010, which measures 6a origin and evolution.

¶ Using the rate 2.737×10^−3^ (1.792×10^−3^∼3.745×10^−3^)±1.51×10^−5^ as a prior, and the resulting MCC tree was shown in Figure 2.

‡ Using the rate 2.062×10^−3^ (1.479×10^−3^∼2.869×10^−3^)±9.94×10^−6^ as a prior, and the MCC tree was shown in Figure S3.

† Directly estimated from the 285 sequences, and the MCC tree was shown in Figure S4.

#### (6) Phylogeography

**Figure 3:**
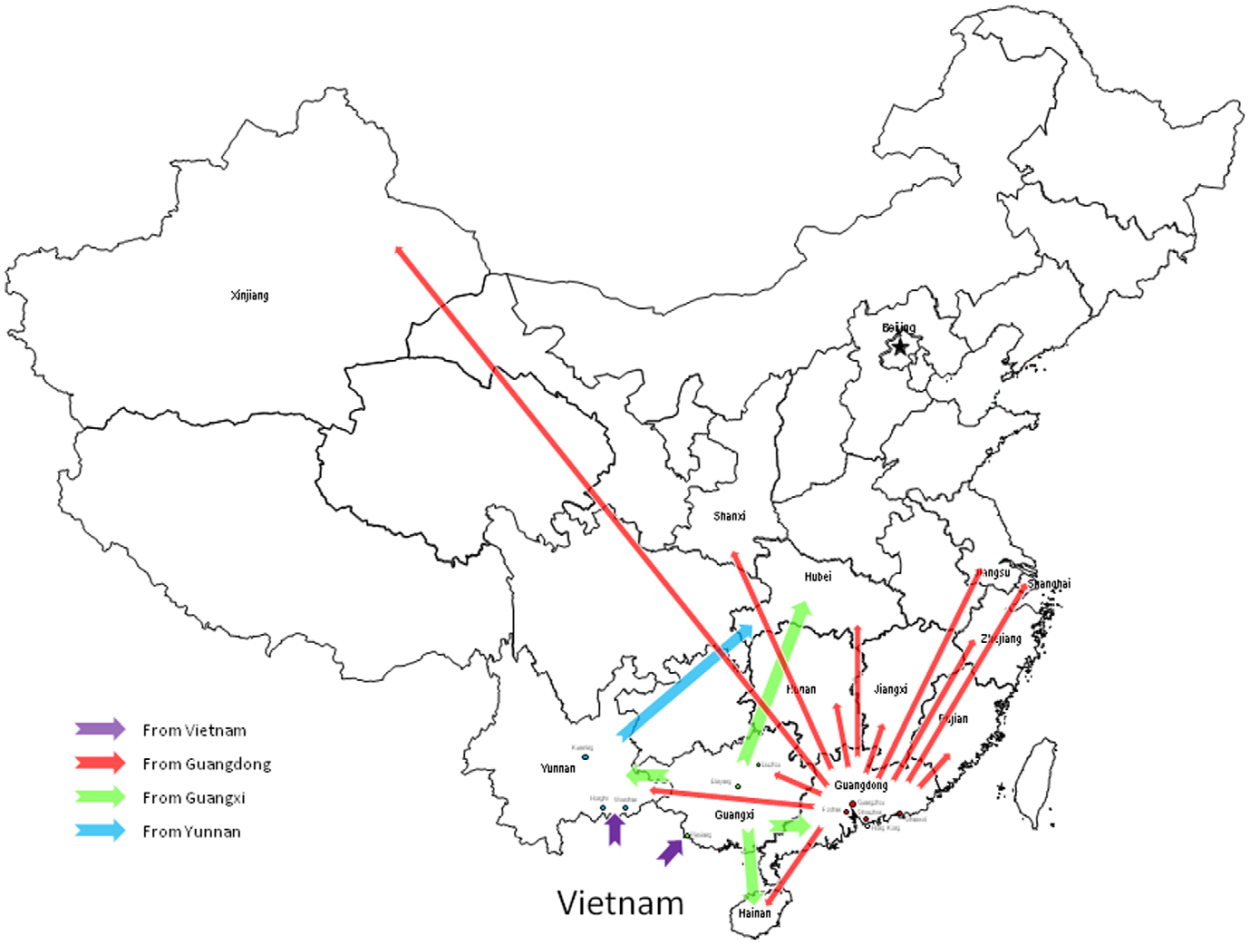
Map of HCV 6a migration in China. With origin from Vietnam, 6a was spread to the neighboring Guangxi and Yunnan Provinces for circulation (shown in pink arrows). From Guangxi, 6a was further spread to the Guangdong, Hainan, Yunnan, and Hubei provinces (shown in green arrows), while from Yunnan, 6a was brought by IDUs to the central part of China (shown in a blue arrow). For unknown reasons, 6a has become a local epidemic in Guangdong and caused infections not only among IDUs, but also among blood donors and patients. As the prevalence is increasing, Guangdong has become the second source region to radiate 6a to other regions (shown in red arrows).

Figure 2 shows the MCC (maximum clade credibility) tree, a phylogeography reconstructed from the trees sampled under the exponential model (Table 4). Seven 6a clusters shown in Figure 1 are all maintained and supported with high posterior probabilities (P≥0.97). Vietnamese isolates are positioned at the base. The base contains the most direct descendents of the earliest 6a common ancestor dated around 1962–1963 (95% CI: 1915–1980), which is thought to be the origin of all 6a strains in China. Upward from the base, there appeared a trend of 6a migration first to Yunnan, to Guangxi, then to Guangdong, and recently from Guangdong to many other regions. Branched from the base, cluster V includes 16 isolates, descended from a common ancestor dated around 1989 (95% CI: 1984–1994). Except two that were from blood donors in Vietnam, all isolates in cluster V were from IDUs in China: 2 from Pingxiang, a city of the Guangxi Province [6], 5 from Honghe and Wenshan, Prefectures of the Yunnan Province [10], and 7 from Wuhan City of the Hubei Province [2]. The former three areas border Vietnam, while the latter is located in central China. Cluster V may illustrate 6a migration from Vietnam to the bordering Guangxi and Yunnan provinces and then transport by IDUs from Yunnan to inland China, over a long distance following a known drug trafficking route [2]. However, this cluster seemed to not link to the main 6a prevalence in the country. The main prevalence appeared to have originated from a different but older ancestor dated around 1978 (95% CI: 1969–1986). From the latter, cluster IV was branched, containing 14 isolates all from IDUs in Pingxiang City of the Guangxi Province [6]. However, cluster IV isolates have not been found elsewhere, possibly due to under-estimation. A similar situation was seen for clusters VI and VII, which link to ancestors dated around 1993 (95% CI: 1989–1995) and 1994 (95% CI: 1990–1996). Cluster VI and VII may represent emerging 6a strains only found among IDUs in Shanwei City. In contrast, cluster II and III isolates showed a wider range of geographic distribution. Derived from a common ancestor dated around 1992 (95% CI: 1987–1994), cluster III contains 42 isolates: 36 from Guangdong and 6 from other provinces (Hunan, Yunnan, Shanghai, Guangxi, and Hubei). Cluster II was descended from an ancestor slightly younger and contains 21 isolates: 17 from Guangdong and 4 from other provinces (Fujian, Zhejiang, Shanxi, and Hubei). Clusters II and III both showed 6a migration inside Guangdong followed by outward movement to other regions. Since most were from blood donors [9], a transition from IDU to non-IDU dissemination is suggested. Remarkably, isolates in cluster I had the largest number size (135 isolates) and showed the widest range of spread (11 provinces). It started from a common ancestor dated around 1990 (95% CI: 1986–1993) and is characteristic of geographic lineages. For example, sequences from three cities of Guangxi Province [3], [6] formed a noticeable group analogous to four smaller neighboring groups that contain sequences mainly from Guangdong and occasionally from other provinces [8], [9]. At the top of the tree, there is a large group containing two additional geographic lineages, one from Bingyang City in the Guangxi Province [6] and the other from Wuhan City in the Hubei Province [2], separated by a bunch of sequences from Guangdong [8], [9]. This large group can be the evidence to support that the IDU networks in cities of Guangxi, Guangdong and Hubei provinces had been exchanged. Although Guangxi is thought to be the origin of 6a in Guangdong, migration from Guangdong back to Guangxi is also suggested. As 6a in Guangdong has become increasingly prevalent, there appeared a recent trend of radiation to other regions. This is brightly portrayed in cluster I, which showed 6a migrations from Guangdong to Yunnan, Fujian, Jiangxi, Hainan, Hunan, Xinjiang, Shanghai, and Shanxi Provinces, and this is associated with blood donors. Similar phylogeographies were also plotted under two other models (Table 4 & Figure S3, S4), and collectively, the 6a migration routes in China were suggested (Figure 3).

#### (7) Migration test

To test for the presence of phylogeographic structures, an additional test was performed to estimate two statistics: AI (Association Index) and PS (Parsimony Score statistic). AI is the sum across all the internal nodes of a phylogeny, which explicitly takes into account the shape of the phylogeny by measuring the imbalance of the internal nodes [18]. The estimation of PS, however, uses a parsimony approach to reconstruct the character states at ancestral nodes and calculate the number of state changes in the phylogeny [19]. According to sampling origins, the 285 sequences were divided into five states (Guangdong, Guangxi, Yunnan, Others, and Vietnam). Correlating the five states with phylogenetic positions showed the AI and PS statistics both strongly rejecting the null hypothesis of panmixis [20]. Therefore, association between lineages and geographic origins was supported.

#### (8) Bayesian Skyline Plots (BSPs)

**Figure 4:**
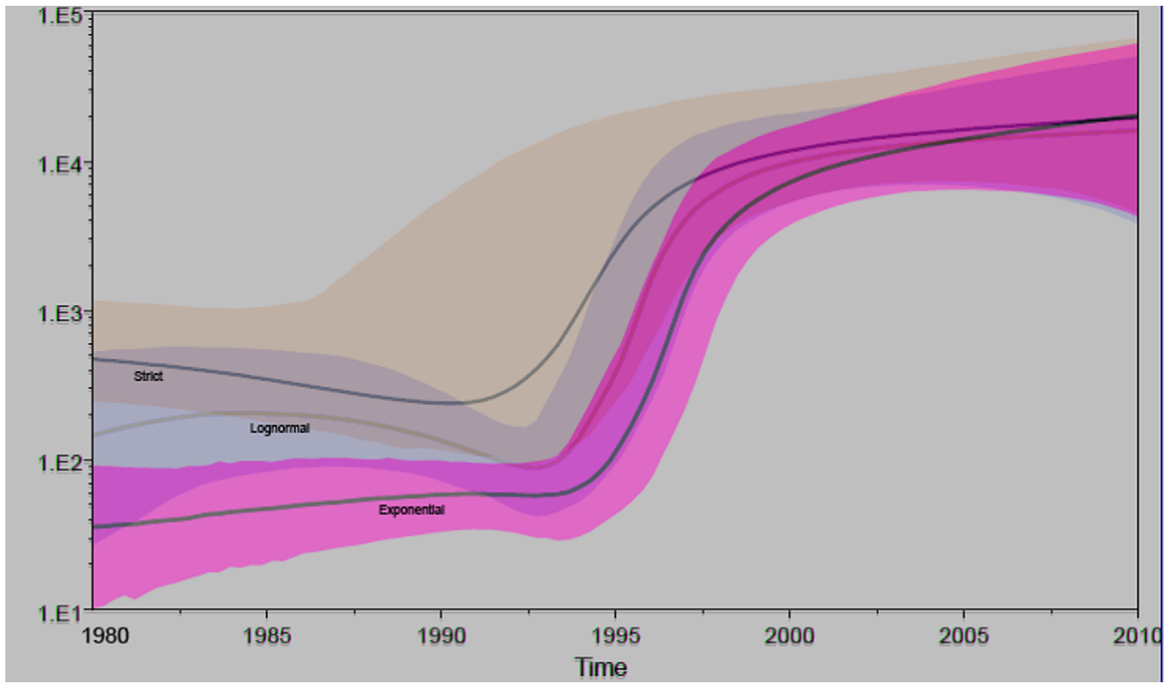
The overlapped BSPs. Three indicated solid lines represent the estimated effective population sizes through time under three combination models: BSP + Uncorrected Exponential, BSP + Uncorrected Lognormal, and BSP + Strict Clock, respectively. The colored areas (pink = Exponential, blue = Lognormal, and yellow = Strict) around the solid lines represent the 95% highest posterior density confidence intervals for these estimates. The vertical ruler on the left scales the effective population size while the horizontal ruler on the bottom measures time.

Figure 4 shows the overlapped BSPs estimated based on three models (Table 4). These are flexible, non-parametric estimates of past changes in population size [11], plotted using the “log” files and “trees” files resulting from three coalescent procedures. Estimated under different models, surprisingly similar patterns of growth were shown. Let's first describe the BSP from the exponential model. A relatively constant population size was maintained from 1980 to around 1994. However, this was breached by a fast exponential growth until 1998. Since 1998, the growth was suddenly slowed and then kept slightly upward till the present. The rapid growth from 1994 to 1998 corresponded to a period when contaminated plasma collection was common in China, which led to about 500,000 blood donors infected with HCV [21], [22] and 300,000 donors infected with HIV [23], [24]. The abrupt slowing around 1998, however, was concurrent to a time point when the Chinese government outlawed the use of paid blood donors, which went into effect that year [25]. Using slightly lower substitution rates (Table 4), the BSPs under two other models showed both the start and end of the rapid growth a little earlier (Figure 4). However, they remained coincident with the period when for-profit plasma collection was common in China, which began in the early 1990 s and was officially forbidden in 1996 [23], [24].

## Discussion

In this study, both E1 and NS5B sequences of HCV were determined among 136 drug users. It resulted that 6a was detected in 70 users (51.5%). The identified 6a predominance is consistent with that from previous reports [2], [3], [6], [10], [26]–[28]. These previous reports have collectively shown increasing 6a predominance accounting for a pooled rate of 36.7% [5]. Similar to that we have recently described, the majority of 6a isolates in this study were classified into three clusters, I, II, and III, with more in cluster III than in cluster II and more in cluster I than in both clusters II and III. In addition, two new clusters, VI and VII, were designated with the isolates exclusively from the present study. Including the reference sequences to indicate their geographic origins showed that isolates in clusters II and III were mainly from Guangdong and occasionally from other regions. Isolates in cluster I were not only from a variety of people (IDUs, patients, and blood donors) in Guangdong, but also from blood donors in 12 other provinces: Guangxi, Hainan, Yunnan, Fujian, Jiangxi, Hunan, Hubei, Jiangsu, Zhejiang, Shanghai, Shanxi, and Xinjiang [2], [3], [6], [8], [9]. With the addition of clusters IV and V, the distribution of 6a in China is illustrated. However, Vietnam was estimated to be the origin of 6a in China. Phylogeographic analysis indicated that the Guangxi Province, which borders Vietnam, could have been the first region to accept 6a for circulation. Migration from Yunnan, which also borders Vietnam, might be equally important, but was only detected among IDUs in limited regions. From Guangxi, 6a could have further spread to the Yunnan, Guangdong, Hainan, and Hubei provinces. However, there is evidence to show that only in Guangdong has 6a become a local epidemic, which has made Guangdong the second source region to disseminate 6a strains to other regions. A map of 6a migration in China is therefore portrayed (Figure 3). The importation of 6a from Vietnam to Guangxi could be attributed to the growing communications among residents living on both sides of the Guangxi-Vietnam border, and more importantly, the free exchange of IDU networks in Guangxi and Southeast Asian countries through the known drug trafficking routes. Apart from this, there are three other possibilities: (1) during the late 1970 s, the Guangdong, Guangxi, and Hainan Provinces accepted over 300,000 refugees from Vietnam, of which a fraction of people might have been infected [9]; (2) 6a may be indigenous in Guangdong and Guangxi, since the two regions are geographically adjacent and historically linked to Vietnam; (3) some 6a strains might have come from Hong Kong, which had an alternative route of 6a introduction [29]. In this study, phylogenetic and phylogeographic analyses both revealed a certain degree of differences among seven 6a clusters and several branches, but not comparable with those existing among the Vietnamese isolates at the tree base. Such differences are not sufficient to validate possibilities (1) and (2). Unfortunately, due to lack of a time-stamped sequence dataset from Hong Kong, at the moment we were unable to test possibility (3). The common ancestor of 6a strains in China was dated around 1978. Concurrently, this was the time point when the China-Vietnam war occurred and when Guangdong was assigned as the first region of China to adopt a free market economy and open door policy. These could be two historical events to facilitate 6a strains imported from Vietnam (via Hong Kong or other Southeastern countries?) to China (first to Guangxi, Guangdong, or Yunnan?) and implanted in an HCV transmission network (via blood donor or IDU network?). During the decades after 1978, Guangdong has led the country in fast economic development and has now become a “World Production Center”. The powerful economy has stimulated a large population of IDUs and the prosperity of drug trafficking and trading, as well as countless visitors for business and pleasure. More importantly, tens of millions of migrants or “farm workers” have been attracted from rural areas to work in the myriad of factories in the Pearl River Delta, a central region of the Guangdong Province. However, due to a lack of optimal working and living conditions, these migrants are more vulnerable to viral infections such as HCV and HIV [23]. Some of them may have also been involved in a thriving illegal sex industry [7] and drugs, while a few may have been HCV carriers from earlier due to previous contaminated blood donations. During holidays and busy farming seasons, these migrants have frequently traveled back and forth between Guangdong and their rural hometowns; 6a strains may have been widely transported.

Blood transfusion was a major risk factor for HCV infection before the virus was identified in 1989. This risk remained high in China even during the mid 1990 s and peaked in 1995. Marked with a scandalous plasma campaign during 1994–1996 in the Henan province, it led to more than 500,000 blood donors that were infected with HCV and 300,000 donors infected with HIV [21]–[25]. Most of the infections were acquired at paid blood donation stations, some operated by local health officials, where many donors' red blood samples were mixed, the plasma extracted, and the pooled red blood cells transfused into the donors. Similar contaminated donations have been also reported in seven other provinces [23]. This disastrous event in China was vitally indicated in the BSPs, which showed a rapid growth in the HCV-infected population size during 1994–1998. In 1998, the Chinese government outlawed the use of paid blood donors to eliminate this risk [25]. Correspondingly, this was reflected in BSPs with an abrupt slowing in the 6a-infected population size, starting from 1998. However, in the poor rural communities a huge infected population has been established, of whom many have now moved with millions of migrant laborers to cities. Through drugs and sex, they still provide a bridge to the general population, and in the BSPs this could be represented with a slightly increasing slope.

## Materials and Methods

### Subjects and specimens

Serum samples were collected in Nov 2010 from 210 drug users retained in a mandatory detoxification center in Shanwei City, Guangdong Province, China. The ethical review committee of the Guangzhou Blood Center had approved this study. Guidelines set by this committee were strictly followed and written informed consent was obtained from each participant. Before bleeding, a questionnaire was answered by each user. Questions included the home address, age, gender, marital status, education, ethnic and geographic origins, histories of blood transfusion and surgery and other exposures, time of starting, the duration, and the method of drug use.

### Anti-HCV and NAT assays

Anti-HCV was assayed as previously described [9]. NAT for HCV RNA was tested on the Procleix TIGRIS system (Novartis Diagnostics, Emeryville, CA, USA) following the manufacturer's protocol.

### DNA amplification, sequencing, and phylogenetic analysis

HCV E1 and NS5B genes were amplified and sequenced as previously described [8], [9]. To confirm multiple HCV infections, amplicons were cloned into a pT-Adv vector (Advantage PCR cloning kit; Clontech). A number of clones were picked and plasmids sequenced [30]. According to HCV subtypes, the resulting sequences were aligned with references using the CLUSTAL_X program. Phylogenetic trees were estimated using the maximum likelihood method under the GTR+I+Γ_6_ substitution model.

### Evolutionary analysis of 6a sequences

For coalescent analyses, time-stamped E1 sequences were further determined among 21 blood donors from Vietnam, 22 blood donors from 12 provinces of China, and 36 IDUs from Liuzhou City in the Guangxi Province [3]. In addition, 139 sequences were retrieved from Genbank. Together with those determined in this study, a total of 285 6a isolates were represented. They all had their sampling dates (from 1992 to 2010) and geographic origins recorded and E1 gene sequences (up to 519 nt) available to compile the dataset. Information from Genbank, from original publications, and from our personal communications were used to verify the sampling dates, to ensure that no sequences were from experimental animals, engineering, no multiple quasispecies were included, nor those that were from single individuals at different time points. Because the change in HCV-infected population size was an object of study, closely related 6a sequences from single epidemic scenarios were not excluded except when they were identical. After alignment and visual corrections, the most appropriate site model was determined using the program jModelTest [12].

Based on the 285 sequences, the evolutionary rate and epidemic history was estimated using the BEAST package [13]. Each MCMC sampling was performed for 200 million states, sampling a tree every 20,000 states. Prior to MCMC, selection was made for (1) site model, (2) demographic model, and (3) clock model. For (1), the GTR+I+Γ model was revealed to be the best among 24 models, based on the criteria from jModelTest. For (2), although the constant size, exponential growth, logistic growth, expansion growth, and BSP all are selectable, a previous study has shown that, excluding BSP, other models consistently performed poorly with HCV sequences [14]. Therefore, we did not test these models but only used the BSP in this study. For (3), we tested all three models: exponential, lognormal, and strict, in combination with BSP respectively, for selecting the best-fitting combination. To analyze the results from the MCMC process, a program Tracer was used [13]. We used it to estimate the marginal likelihoods of the trace, check the trace distribution for convergence, and determine if sufficient ESS (>200) was achieved. We also used it to compute a BF for each pair of models for selecting the statistically best. In addition, we used it to reconstruct BSP to show the epidemic history of HCV. Through the MCMC process, the BSP generalizes skyline plots from the targeted sequence dataset, then samples the distribution of the generalized skyline plots, and combines the plots to yield a posterior distribution of effective population size through time. Since credibility intervals are provided for effective population size at every point in time, from the present back to the most recent common ancestor of the sampled sequences, a past history of HCV-infected population size is inferred [11].

After discarding 10% of burn-in, a time-scaled phylogeny was estimated from a posterior distribution of trees that were generated under a given model combination. We used the TreeAnnotator program to construct this phylogeny, which best summarizes the set of credible trees and is called the MCC tree. Since a molecular clock was used in the MCMC process, the branch lengths and node height of the tree are in units of years. Phylogeographic structure was then displayed using the FigTree program, and clades and lineages were colored according to their geographic origins [13].

In addition, the Befi-BaTS program was used to test for the presence of statistically significant phylogeographic structure (www.lonelyjoeparker.com/?tag=befi-bats). This was done through a parametric bootstrap process, which randomizes the association a large number of times, calculates statistics from each randomization, and provides a null distribution of the statistics with 0.95 credible intervals. Against this level the observed statistics are compared. Here, the null hypothesis of panmixis assumes that there is no correlation between phylogeny and taxa location [19], and the randomization is performed against a series of tree-shaped statistics. We performed randomizations across a posterior distribution of trees generated from the MCMC process under a given coalescent model. During the bootstrapping process, the phylogenetic uncertainties were correctly incorporated and phylogeographic structure tested [20].

### Nucleotide sequence accession numbers

The sequences reported in this paper have been deposited in GenBank with the following accession numbers: JF720958-JF721322.

## Supporting Information

Figure S1 **Subtype 1b phylogenies estimated from (A) E1 and (B) NS5B region sequences, corresponding to H77 nucleotide positions of 869–1289 and 8276–8615, respectively.** Subtype 1a sequence M62321 was used as an outgroup. Green pies label sequences from our previous studies [8], [9]. Red and yellow pies label sequences from this study, in which yellow pies mark isolates from IDUs with multiple HCV infections. Sequences without pies were retrieved from Genbank. In each tree, six rectangles highlight the further classification of 1b isolates into A, B, C, D, E, and SW clusters. Scale bar represents 0.05 nucleotide substitutions per site. Bootstrap support values are shown in italics.
(TIF)

Figure S2 **Subtype 3a phylogenies estimated from (A) E1 and (B) NS5B region sequences, corresponding to H77 nucleotide positions of 869–1289 and 8276–8615, respectively.** Subtype 3b sequence D49374 was used as an outgroup. Two geographic clusters were shown with sequences from Hubei [2] and Yunnan [10] provinces to compare with a geographic cluster from Guangdong. Otherwise, all labels are the same as those described in the Figure 1 legend.
(TIF)

Figure S3 **MCC tree estimated under the model of BSP + Uncorrelated Lognormal (** **Table 4** **).** See Figure 2 for legend.
(TIF)

Figure S4 **MCC tree estimated under the model of BSP + Strict Clock (** **Table 4** **).** See Figure 2 for legend.
(TIF)
