# Supplementary figures and images for: HCV 6a Prevalence in Guangdong Province Had the Origin from Vietnam and Recent Dissemination to Other Regions of China: Phylogeographic Analyses
Source: PLoS One. 2012 Jan 9;7(1):e28006. doi: 10.1371/journal.pone.0028006 (PMC3253785; doi:10.1371/journal.pone.0028006)

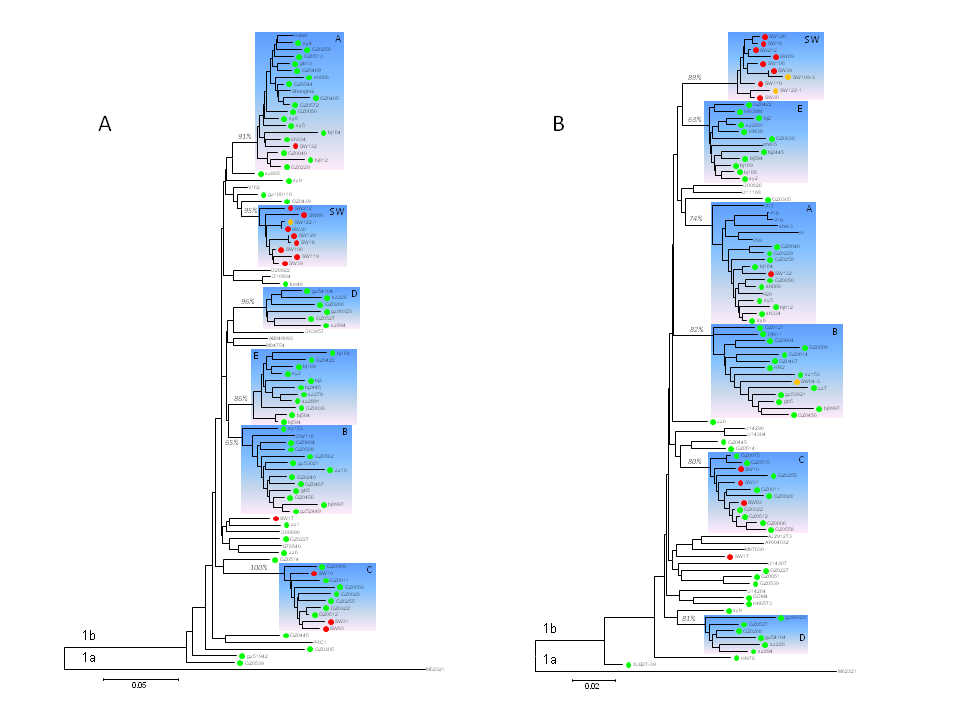

Supplement: Figure S1 — Subtype 1b phylogenies estimated from (A) E1 and (B) NS5B region sequences, corresponding to H77 nucleotide positions of 869–1289 and 8276–8615, respectively. Subtype 1a sequence M62321 was used as an outgroup. Green pies label sequences from our previous studies [8], [9]. Red and yellow pies label sequences from this study, in which yellow pies mark isolates from IDUs with multiple HCV infections. Sequences without pies were retrieved from Genbank. In each tree, six rectangles highlight the further classification of 1b isolates into A, B, C, D, E, and SW clusters. Scale bar represents 0.05 nucleotide substitutions per site. Bootstrap support values are shown in italics. (TIF) [file pone.0028006.s001.tif]

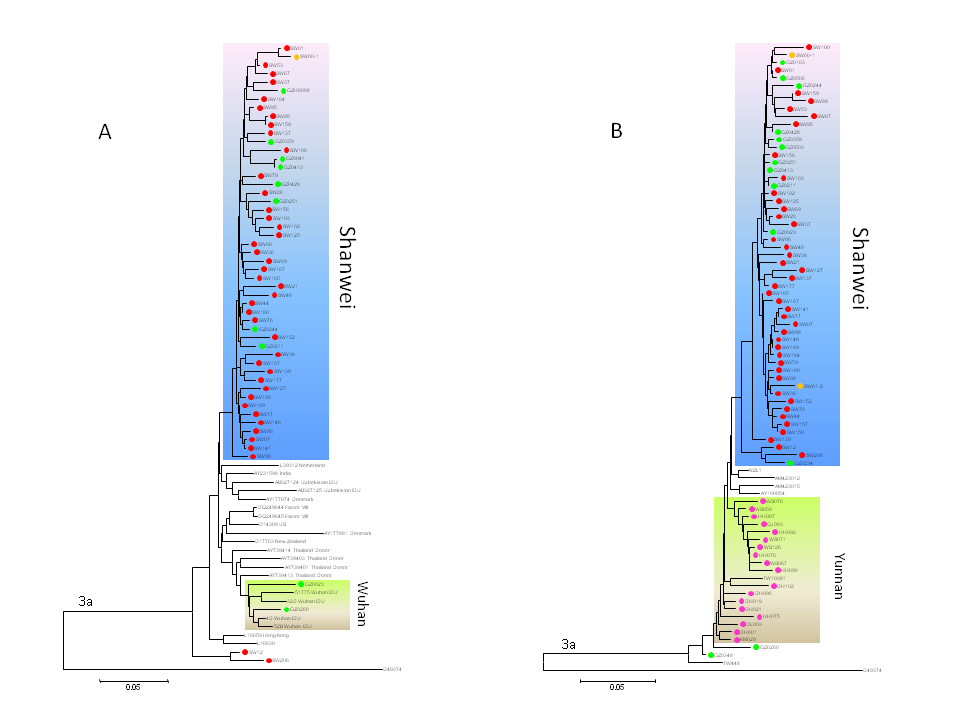

Supplement: Figure S2 — Subtype 3a phylogenies estimated from (A) E1 and (B) NS5B region sequences, corresponding to H77 nucleotide positions of 869–1289 and 8276–8615, respectively. Subtype 3b sequence D49374 was used as an outgroup. Two geographic clusters were shown with sequences from Hubei [2] and Yunnan [10] provinces to compare with a geographic cluster from Guangdong. Otherwise, all labels are the same as those described in the Figure 1 legend. (TIF) [file pone.0028006.s002.tif]

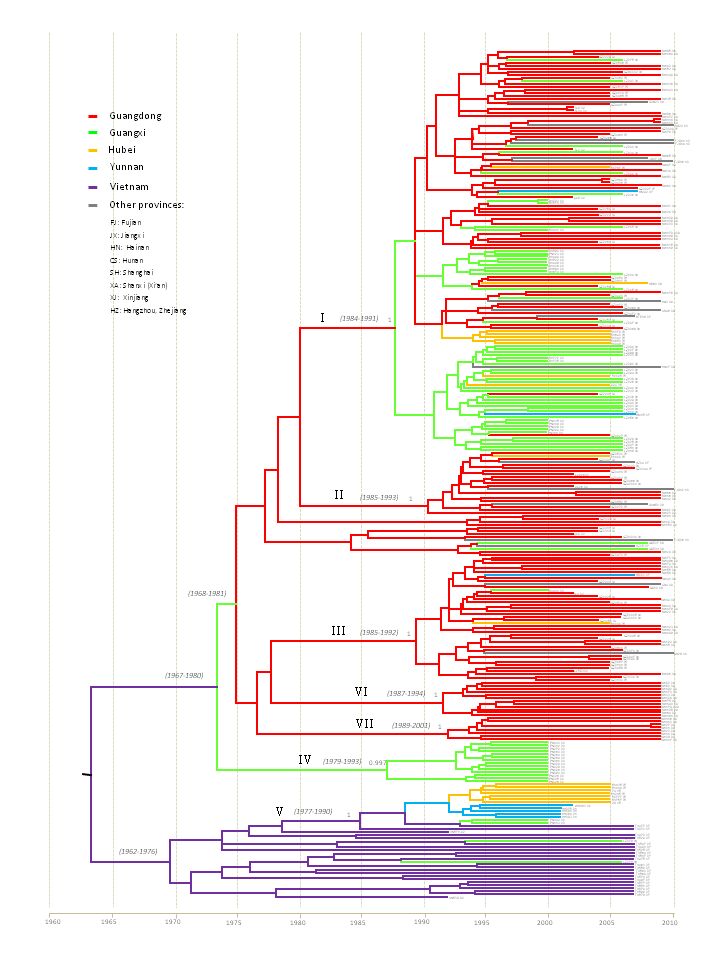

Supplement: Figure S3 — MCC tree estimated under the model of BSP + Uncorrelated Lognormal ( Table 4 ). See Figure 2 for legend. (TIF) [file pone.0028006.s003.tif]

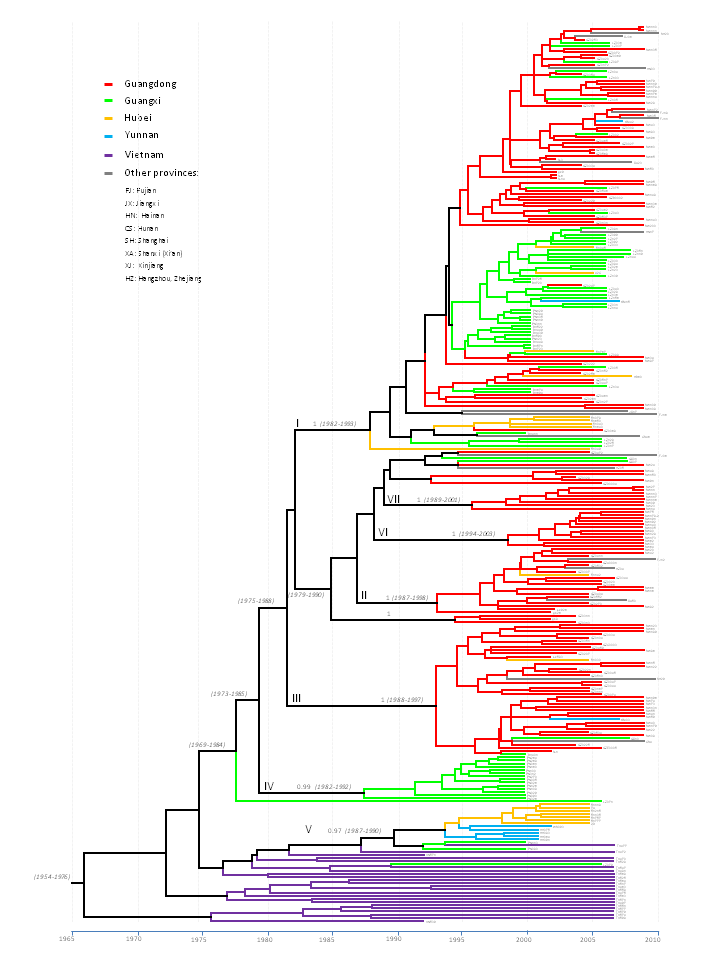

Supplement: Figure S4 — MCC tree estimated under the model of BSP + Strict Clock ( Table 4 ). See Figure 2 for legend. (TIF) [file pone.0028006.s004.tif]
